# Time-split validation and cross-platform transferability of an explainable MALDI-TOF MS machine-learning model for carbapenem-resistant *Klebsiella pneumoniae* screening

**DOI:** 10.3389/fmicb.2026.1985869

**Published:** 2026-10-28

**Authors:** Kun Li, Xue Yang, Long Li, Jie Wang

**Affiliations:** Department of Clinical Laboratory Medicine, Suining Central Hospital, Suining, Sichuan, China

**Keywords:** carbapenem resistance, cross-platform transferability, *Klebsiella pneumoniae*, machine learning, MALDI-TOF MS, time-split validation

## Abstract

MALDI-TOF MS-based machine learning may shorten the time to detection of carbapenem-resistant *Klebsiella pneumoniae* (CRKP), yet performance estimates often rest on random splits and spectrum-level evaluation, and transferability across instruments is rarely tested. In a laboratory-information-system-audited single-center cohort of 2,399 *K. pneumoniae* isolates (363 CRKP; 2,836 VITEK MS spectra; 2024–2026), we trained a LightGBM classifier on 1,766 isolates from 2024 to 2025 and evaluated it on 633 isolates from 2026. The out-of-fold area under the ROC curve (AUC) was 0.909 (95% CI 0.885–0.932); on the 2026 cohort the calibrated AUC was 0.833 (0.776–0.885), with an expected calibration error of 0.024 and a Brier score of 0.068. Thresholds derived independently from the training cohort gave a sensitivity of 0.493 on the 2026 cohort at both the Youden and the NPV-oriented threshold, with an observed NPV of 0.935; the higher sensitivity obtained with thresholds chosen on the 2026 cohort itself was not reproducible. Applying the model unchanged to 1,190 isolates from three other platforms (Autof, Bruker, Quantof) resulted in loss of essentially all discriminative ability (AUC 0.450–0.543); because those datasets also differ in population and label definition, the loss cannot be attributed to platform alone. SHAP analysis identified a dominant m/z 4517.5–4519.5 cluster. A deployable offline tool reproduced the audited performance (AUC 0.826, ECE 0.022), and a monitoring analysis on 564 spectra drawn from the same period was consistent with it. The model is not a stand-alone screening test: deployment is limited to the platform on which it was developed and validated, and requires ongoing monitoring. The labeled dataset is deposited in MassIVE (MSV000103080) for independent benchmarking.

## 1 Introduction

CRKP is a WHO priority-1 critical pathogen (Tacconelli et al., 2018). In endemic settings, carbapenem resistance rates exceed 75% in some Asian intensive care units, and bloodstream CRKP infection carries an attributable mortality above 40% (Nordmann et al., 2011; Pitout et al., 2015). Phenotypic antimicrobial susceptibility testing (AST) takes 48–72 h after culture, so patients receive empirical treatment during the highest-risk window. Among patients with septic shock, delay in effective antimicrobial administration was associated with reduced survival. For each hour of delay during the first 6 hours after hypotension onset, survival fell by an average of 7.6% (Kumar et al., 2006). Delays of this order are common while phenotypic susceptibility results are pending.

MALDI-TOF MS identifies bacteria from routine spectra within minutes (Seng et al., 2009). Beyond species identification, resistance-associated spectral signatures have been reported (Weis et al., 2022; Yu et al., 2023; Singhal et al., 2015). Machine-learning (ML) classifiers can detect resistance directly from spectra (Tang et al., 2021; Yegorov et al., 2024; Tran et al., 2021), including carbapenem resistance in *K. pneumoniae* (Wang et al., 2022; Cheon et al., 2025; Gato et al., 2023; Cordovana et al., 2018), and MALDI-TOF MS is now established in routine clinical microbiology (Bizzini et al., 2010; Calderaro and Chezzi, 2024); proteomic and resistance profiles of *K. pneumoniae* clinical isolates have also been characterized on this platform (Marzouk et al., 2024). Three issues remain unresolved across this literature. First, reported performance usually rests on random splits of a single cohort. Because training and test spectra then share acquisition periods, batches, and instrument settings, such splits overestimate real-world performance. Estimates are inflated further when evaluation is performed at the spectrum rather than the isolate level, because replicate spectra of one isolate are correlated. Time-split (i.e., temporal) validation, in which the test set is acquired after the training period, remains less common (Varoquaux et al., 2017). Second, transferability across instruments is rarely tested, even though MALDI-TOF mass accuracy, resolution, and laser response differ between platforms (Cuénod et al., 2023; Weis et al., 2020). Where cross-platform performance has been examined, platform has usually varied together with population and label definition (Cai et al., 2025). Whether a fixed, already deployed model can be reused on a second instrument has therefore not been answered directly. Third, few studies describe how a model is monitored or retrained once it enters routine use (Gama et al., 2014), even though drift in acquisition conditions is expected.

We addressed these issues within a single study design. In a laboratory-information-system (LIS)-audited cohort of 2,399 *K. pneumoniae* isolates with 2,836 VITEK MS spectra, we trained a LightGBM classifier on isolates acquired during 2024–2025 and validated it on isolates acquired in 2026. We report calibration and derive decision thresholds from the training cohort rather than from the validation cohort. The same model was then applied without modification to 1,190 isolates measured on three other MALDI-TOF platforms (Autof, Bruker, Quantof). We also built a calibrated offline executable with configurable decision thresholds and a companion monitoring tool, and specified how the model should be retrained once in routine use. The labeled spectra and per-spectrum metadata are deposited in MassIVE (MSV000103080) as a benchmark dataset, so that the estimates reported here can be checked independently.

## 2 Materials and methods

### 2.1 Study design and isolates

This retrospective study was conducted at Suining Central Hospital, a tertiary-care hospital in Sichuan, China. We included consecutive, non-duplicate *K. pneumoniae* clinical isolates with routine MALDI-TOF spectra and AST results. Isolates were classified by routine meropenem susceptibility testing, interpreted with Clinical and Laboratory Standards Institute (CLSI M100) criteria (Clinical and Laboratory Standards Institute, 2022): carbapenem-resistant *K. pneumoniae* (CRKP), meropenem MIC ≥ 4 mg/L or disk-diffusion zone diameter ≤ 19 mm; carbapenem-susceptible *K. pneumoniae* (CSKP), meropenem MIC ≤ 1 mg/L or zone diameter ≥ 23 mm. Susceptibility was determined in routine practice by either VITEK 2 MIC (bioMérieux, Marcy-l’Étoile, France) or disk diffusion, according to the method used in the reporting laboratory on the day of testing; disk diffusion was the more frequent route in this cohort. No isolate with an intermediate meropenem result was present in the cohort, so the susceptible and resistant groups are unaffected by the treatment of the intermediate category. All labels were reconciled against the LIS before analysis. During this reconciliation, an intermediate data export was found to list 287 isolates from the CSKP group together with a carbapenemase (KPC) enzyme-type entry although their meropenem phenotype was susceptible. Because that combination is unusual, the records were checked against the primary LIS entries and the enzyme-type entries were traced to a data-saving error in the intermediate export; they had no counterpart in the primary records and were removed. The affected isolates were retained in the CSKP group on the basis of their phenotype, which is the reference standard used throughout this study, and no isolate changed group as a result. After correction, no isolate in the dataset carries a carbapenemase type together with a susceptible meropenem phenotype. The study was approved by the hospital ethics committee (protocol KYLLKS20240192); informed consent was waived for this retrospective study of archived isolates and de-identified data.

### 2.2 MALDI-TOF MS acquisition and preprocessing

Isolates were measured on a VITEK MS (bioMérieux, Marcy-l’Étoile, France) using the standard direct-smear protocol in linear positive mode over 2,000–12,000 Da. Only spectra with a species-identification confidence of ≥90% were retained. The research pipeline applied SNIP baseline correction, FFT-based alignment (500 ppm), total-ion-current (TIC) normalization with square-root transform, and 1-Da binning over 2,000–12,000 Da. Binning at 1-Da resolution defines 10,001 grid points; one zero-variance grid point was removed, leaving 10,000 features per spectrum. Spectra with fewer than 20 non-zero bins were excluded. All preprocessing parameters were fixed before validation. Analyses were performed in Python 3 with NumPy, SciPy, scikit-learn, and LightGBM (Pedregosa et al., 2011; Harris et al., 2020; Virtanen et al., 2020; Ke et al., 2017).

### 2.3 Model development and calibration

A LightGBM classifier was trained on isolates acquired during 2024–2025 (*n* = 1,766; CRKP 288, CSKP 1,478; 2,109 spectra) with class weighting. The analysis pipeline was specified as follows. First, the grouping unit for cross-validation was the isolate: all replicate spectra belonging to one specimen identifier were held in the same fold, so that no isolate contributed spectra to more than one fold. Second, the time split was defined at the isolate level: an isolate entered the 2026 cohort if any of its spectra had been acquired in 2026. We verified that the two cohorts share no isolate, and that all test acquisitions are later than all training acquisitions. Third, hyperparameters were selected by Optuna (Akiba et al., 2019) using the 5-fold GroupKFold out-of-fold area under the ROC curve (AUC) computed within the training cohort only; the 2026 cohort was not used during optimization. Fourth, probabilities were calibrated with CalibratedClassifierCV (5-fold, isotonic) fitted on the training cohort only and applied unchanged to the 2026 cohort. Isolate-level predictions were computed as the mean of replicate spectra, and bootstrap confidence intervals were computed at the isolate level.

Discrimination was quantified by the AUC with 2,000-bootstrap 95% confidence intervals (CIs). Calibration was assessed by the expected calibration error (ECE; 10 bins) and the Brier score. Decision thresholds were handled in two ways, and the distinction is important for interpretation. (i) Descriptive operating points were computed directly on the 2026 cohort. These were the fixed 0.5 threshold, the Youden threshold, and thresholds achieving a positive predictive value (PPV) ≥ 99% or a negative predictive value (NPV) ≥ 95%. They are reported as descriptive because the validation cohort contributed to their selection. (ii) Independently derived thresholds were obtained from the training cohort alone, using the same 5-fold GroupKFold grouping by isolate; within each fold a LightGBM model with the selected hyperparameters was fitted and wrapped in an isotonic calibrator, and the resulting out-of-fold probabilities were averaged within isolate. The Youden, PPV ≥ 99% and NPV ≥ 95% thresholds were then read from these training-only out-of-fold predictions and applied unchanged to the 2026 cohort. SHAP (TreeExplainer) was used for global feature importance (Lundberg and Lee, 2017).

### 2.4 Cross-platform transferability

The trained research-pipeline model was applied without modification to an external multi-instrument dataset of 1,190 *K. pneumoniae* isolates (Zenodo 15273330; Cai et al., 2025) measured on Autof, Bruker, and Quantof platforms. Labels were defined by the original authors (KPC vs. non-KPC; CRKP vs. CSKP; NDM vs. non-NDM). External spectra were converted to centroid spectra and binned on the same 1-Da grid to match the training representation. AUC and calibration slope were computed for four label cohorts. These external cohorts differ from the training data in platform, bacterial population and, for some cohorts, label definition. The analysis therefore measures transferability to the external datasets as a whole, and does not isolate the contribution of the instrument platform. Pearson correlations between the binned feature vectors of the same isolate measured on two platforms were computed for isolates represented on more than one platform (299 pairs from 231 isolates).

### 2.5 Deployment implementation and monitoring

For the offline tool, features were encoded by integer m/z binning over 2,000–12,000 Da (10,001 grid points) with TIC normalization, matching the deployed software. The pipeline (standard scaling, variance filtering, LightGBM) retains 9,554 bins after variance filtering and was retrained on the identical training cohort with nested 5-fold calibration (sigmoid) and evaluated on the same 2026 cohort. The two pipelines therefore differ in three respects. The research pipeline applies a square-root transform before binning, keeps 10,000 bins after variance filtering, and uses isotonic calibration; the deployed tool omits the transform, keeps 9,554 bins, and uses sigmoid calibration. Both are reported on the same cohort, so that the effect of these choices is visible. The tool (CRKP-CSKP-Predictor) and a monitoring application (CRKP-Model-Monitor) were compiled into standalone Windows executables. Decision thresholds and alert targets are configurable via a JSON file. The deployed pipeline was also run in routine operation, using the Youden threshold, on 564 spectra from 551 isolates. All 551 isolates and all 564 spectra belong to the 633-isolate, 727-spectrum 2026 cohort, and their acquisition dates fall in the same period (January–July 2026). The monitoring run therefore re-processes a subset of the validation cohort (551 of 633 isolates; 564 of 727 spectra) rather than an independent sample. It is reported as a reproducibility check of the deployed pipeline, and not as an independent estimate of performance.

### 2.6 Monitoring and retraining strategy

Because MALDI-TOF acquisition conditions drift over time and a model validated on one platform is not expected to transfer to another, we define how the deployed tool is maintained. The companion monitoring application recomputes recall, specificity, accuracy and MCC against confirmed susceptibility results as they accumulate. It raises an alert when any of four pre-set targets is missed: recall 0.55, specificity 0.90, accuracy 0.85, and MCC 0.50. Those targets sit at the operating point observed on the 2026 monitoring set, so an alert means performance has fallen below the validated level. On our protocol, retraining takes place every 6 months with newly acquired spectra, and immediately whenever an alert is raised. Retraining reuses the identical feature encoding and model class; confirmed isolates from the new period are added to the training pool; hyperparameters are re-optimized by Optuna under the same isolate-level GroupKFold protocol; the calibrator is refitted on the updated pool; and a candidate model is redeployed only if it reproduces or exceeds the AUC of the current model on a held-out recent period. Each deployed version records its training window, so that the performance of a given version can be attributed to the data it saw.

### 2.7 Statistical analysis

Bootstrap CIs for AUC used 2,000 resamples at the isolate level, and ECE and the Brier score were computed the same way. All reported test-set metrics therefore refer to isolates rather than individual spectra; replicate spectra from one isolate were averaged before evaluation.

## 3 Results

### 3.1 Cohort and quality control

After LIS reconciliation, the cohort comprised 2,399 isolates (CRKP 363, CSKP 2,036) with 2,836 spectra passing quality control (QC) (Figure 1). The training cohort (2024–2025 acquisition) contained 1,766 isolates (CRKP 288, CSKP 1,478) and 2,109 spectra. The time-split test cohort (2026 acquisition) comprised 633 isolates (CRKP 75, CSKP 558) and 727 spectra. Test isolates were all acquired after the training period, and no isolate appeared in both sets. CRKP prevalence was 16.3% in training and 11.8% in the time-split test.

**Figure 1:**
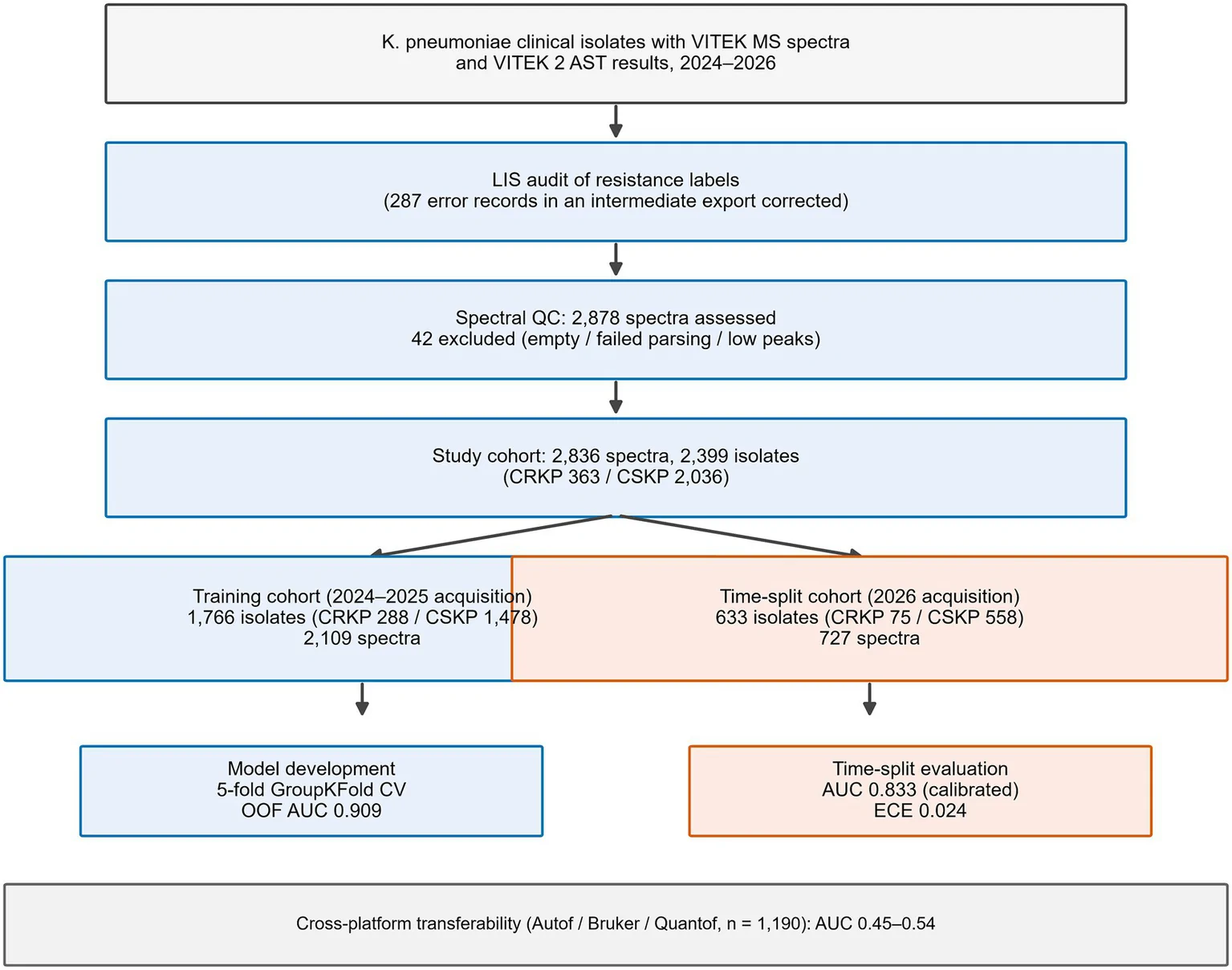
Study cohort and analysis design. LIS, laboratory information system; AST, antimicrobial susceptibility testing; CV, cross-validation; ECE, expected calibration error.

### 3.2 Time-Split validation

The five-fold out-of-fold AUC on the training cohort was 0.909 (95% CI 0.885–0.932). On the 2026 time-split cohort, the uncalibrated AUC was 0.839 (0.781–0.893). After nested cross-validation calibration, the AUC was 0.833 (0.776–0.885), with an ECE of 0.024 and a Brier score of 0.068 (Table 1; Figure 2).

**Table 1:** Performance of the LightGBM model on the 2026 time-split cohort (*n* = 633 isolates; CRKP 75, CSKP 558).

Metric	Estimate (95% CI)
AUC, 2026 cohort (calibrated)	0.833 (0.776–0.885)
ECE	0.024
Brier score	0.068
Descriptive: fixed 0.5 threshold, sensitivity / specificity	0.347/0.996
Descriptive: fixed 0.5 threshold, PPV / NPV	0.929/0.919
Descriptive: Youden threshold 0.08, sensitivity / specificity	0.693/0.864
Descriptive: PPV ≥ 99% (≥0.963), sensitivity / specificity	0.107/1.000
Descriptive: NPV ≥ 95% (≤0.083), sensitivity / specificity	0.667/0.869
Training-derived Youden 0.154, sensitivity / specificity	0.493/0.962
Training-derived NPV ≥ 95% (0.199), sensitivity / specificity	0.493/0.975
Training-derived NPV ≥ 95% (0.199), observed NPV	0.935
Training-derived PPV ≥ 99% (0.885), sensitivity / specificity	0.213/0.998

The first block reports discrimination and calibration; the second block reports descriptive operating points whose thresholds were selected on this cohort; the third block reports operating points whose thresholds were derived from the training cohort alone and applied unchanged. Confidence intervals are given where they were computed (AUC). AUC, area under the ROC curve; ECE, expected calibration error; PPV, positive predictive value; NPV, negative predictive value; CI, confidence interval.

**Figure 2:**
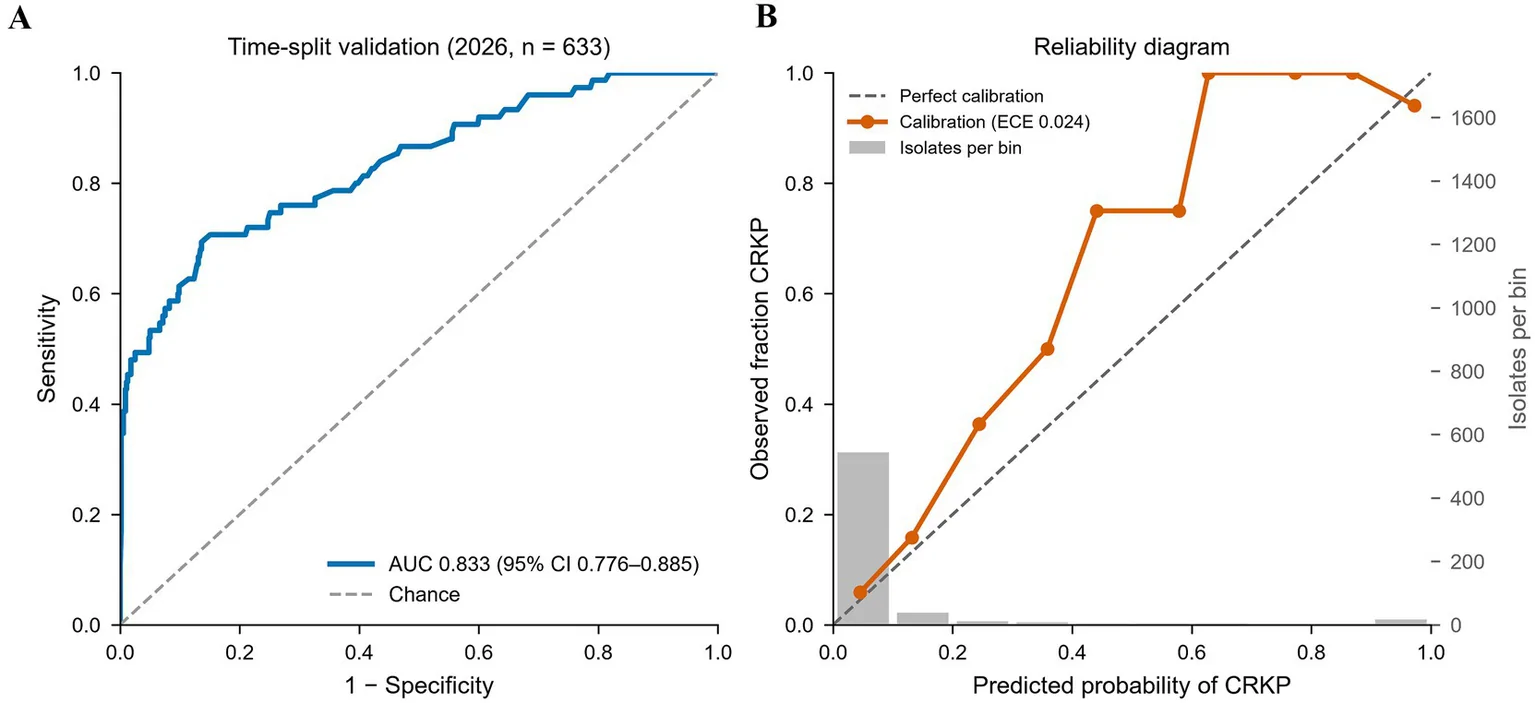
Time-split validation of the LightGBM model on the 2026 cohort (*n* = 633 isolates). **(A)** ROC curve with the area under the curve (AUC; 95% CI). **(B)** Reliability diagram. Predicted probabilities were grouped into 10 equal-width bins; the grey bars show the number of isolates in each bin (right axis), and the diagonal indicates perfect calibration. The expected calibration error is 0.024. AUC, area under the ROC curve; CI, confidence interval.

Threshold-dependent performance was assessed in two ways, and the two differ substantially. When thresholds were chosen on the 2026 cohort itself (descriptive operating points), the fixed 0.5 threshold gave a sensitivity of 0.347 and a specificity of 0.996 (PPV 0.929, NPV 0.919); the Youden threshold (0.08) yielded a sensitivity of 0.693 and a specificity of 0.864. A threshold achieving PPV ≥ 99% (≥0.963) reached a specificity of 1.000 at a sensitivity of 0.107. A threshold meeting NPV ≥ 95% (≤0.083) attained a sensitivity of 0.667 at a specificity of 0.869. These figures are descriptive, because the 2026 cohort contributed to their selection.

When thresholds were instead derived from the training cohort alone (1,766 isolates; out-of-fold AUC 0.905 after per-fold isotonic calibration, versus 0.909 uncalibrated) and applied unchanged to the 2026 cohort, the picture changed. Fixing the Youden threshold on the training cohort (0.154) gave a sensitivity of 0.493 and a specificity of 0.962 on the 2026 cohort, against 0.693 and 0.864 when the threshold was chosen *post hoc* on that cohort. The threshold targeting an NPV of 95% (0.199) gave a sensitivity of 0.493 and a specificity of 0.975, and its observed NPV in the 2026 cohort was 0.935, short of the 95% target. For PPV ≥ 99% (0.885), sensitivity was 0.213 and specificity 0.998 (Supplementary Table S2). The higher sensitivity obtained with thresholds selected on the validation cohort was therefore not reproducible when the threshold was fixed in advance.

### 3.3 Explainability

SHAP global importance was dominated by a three-bin cluster centered at m/z 4518.5 Da (4517.5, 4518.5, 4519.5), followed by m/z 2759.5/2760.5 and 5938.5 (Figure 3). The same cluster carried the most weight in the training and the time-split validation cohorts. Its molecular identity is not yet confirmed.

**Figure 3:**
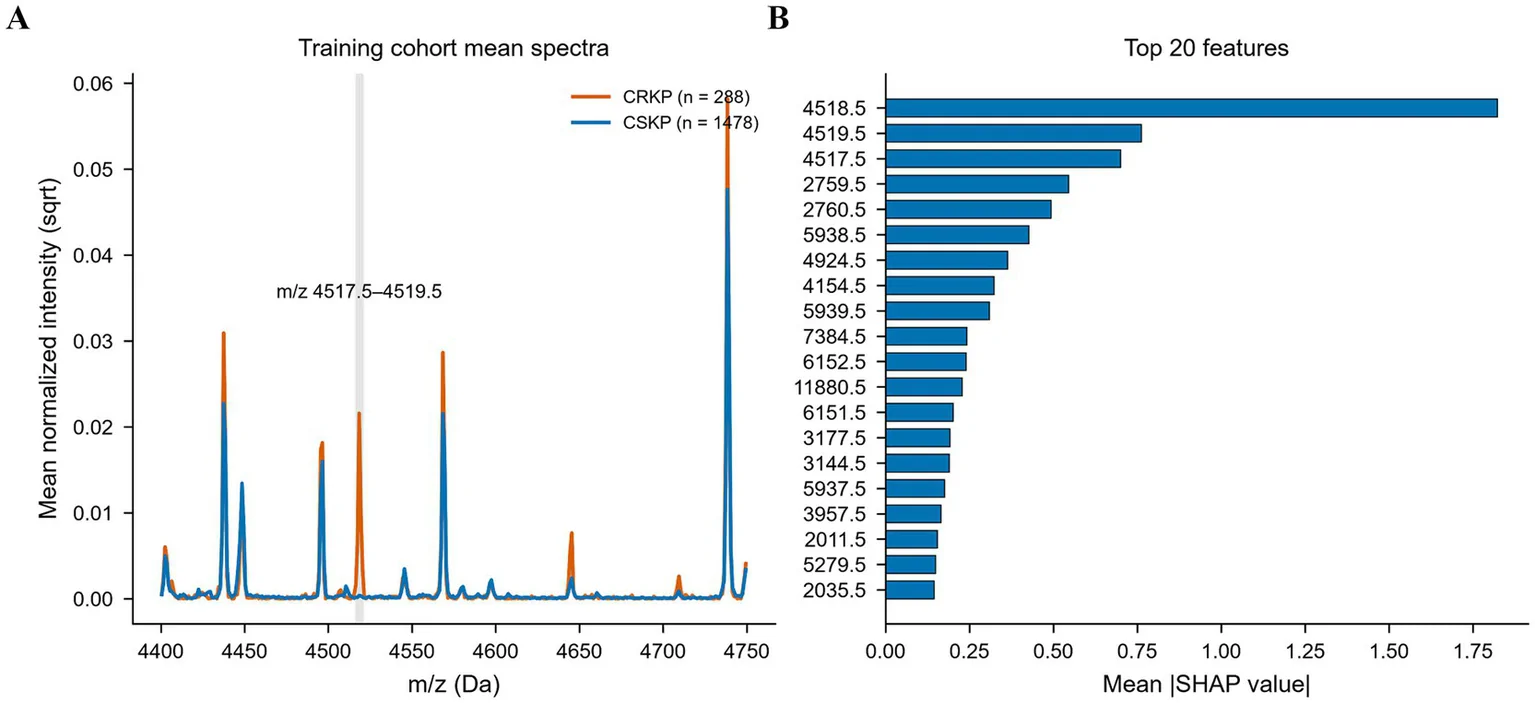
Explainability. **(A)** Mean spectra of CRKP (*n* = 288) and CSKP (*n* = 1,478) isolates in the training cohort around the *m/z* 4517.5–4519.5 cluster. Spectra were total-ion-current normalized and binned at 1 Da; the curve shows the mean intensity per bin within each group. **(B)** Top 20 features ranked by mean absolute SHAP value, computed with TreeExplainer on the trained LightGBM model and averaged across the training cohort. SHAP, Shapley additive explanations.

### 3.4 Cross-platform transferability

Applied without modification to external data from other platforms, the model lost essentially all discriminative ability (Table 2; Figure 4). AUC was 0.540 (0.505–0.576) for Autof KPC (*n* = 997), 0.530 (0.488–0.570) for Bruker KPC (*n* = 821), 0.543 (0.506–0.579) for Autof CRKP/CSKP (n = 920), and 0.450 (0.377–0.520) for Quantof NDM (*n* = 237). Calibration slopes were near zero (0.02–0.03), so the predicted probabilities carried no information. Correlations between the binned feature vectors of the same isolates measured on two platforms were moderate to high rather than absent (median Pearson r 0.89 for Autof versus Bruker and 0.64 for the comparisons involving Quantof; 299 isolate pairs from 231 isolates), so the loss of transfer is not explained by gross dissimilarity of the spectra. Because these four cohorts also differ in bacterial population and, for the KPC and NDM analyses, in the classification endpoint, the results establish that the model does not transfer to these external datasets; they do not establish how much of the loss is attributable to the instrument platform as opposed to population or label differences.

**Table 2:** Cross-platform application of the trained model to the external dataset.

Label definition	Platform	*n*	AUC (95% CI)	Calibration slope
KPC vs. non-KPC	Autof	997	0.540 (0.505–0.576)	0.02
KPC vs. non-KPC	Bruker	821	0.530 (0.488–0.570)	0.03
CRKP vs. CSKP	Autof	920	0.543 (0.506–0.579)	0.02
NDM vs. non-NDM	Quantof	237	0.450 (0.377–0.520)	0.02

The four rows are subsets of the 1,190-isolate external dataset (Zenodo 15273330) distinguished by instrument platform and by the label definition used by the original authors; the subsets overlap, because the same isolates were analysed more than once under different label definitions. AUC values are given with 95% bootstrap confidence intervals. Because the subsets differ in platform, population and label definition, the results show failure of transfer to these datasets and do not isolate the contribution of platform.

**Figure 4:**
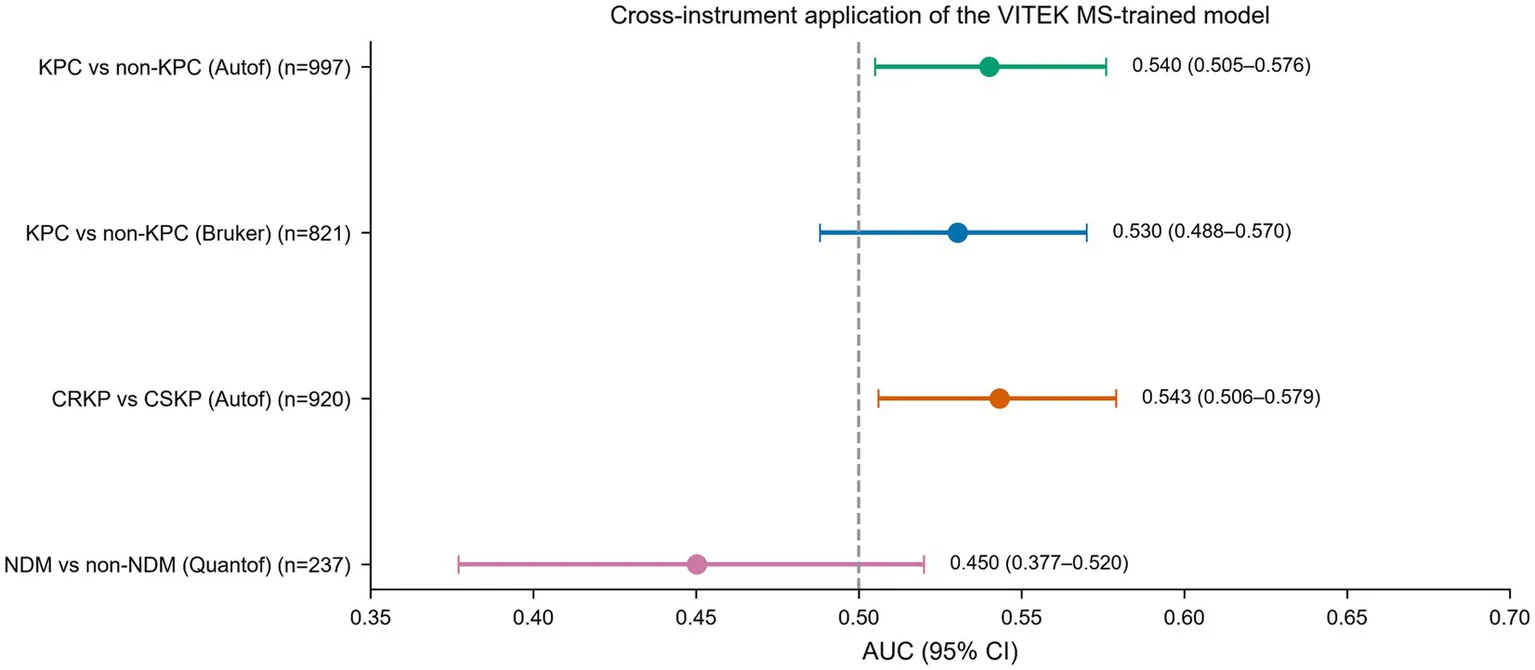
Cross-platform application of the VITEK MS-trained model to external cohorts measured on Autof, Bruker, and Quantof instruments. The four panels correspond to four label definitions provided by the original authors. These are KPC versus non-KPC on Autof (*n* = 997) and on Bruker (*n* = 821), CRKP versus CSKP on Autof (*n* = 920), and NDM versus non-NDM on Quantof (*n* = 237). Because these cohorts differ in platform, population, and label definition, the panels are presented together to show that the model did not transfer to any of them, and not to isolate the effect of platform. Error bars are 95% bootstrap CIs; the dashed line indicates chance (AUC 0.5).

### 3.5 Deployment and monitoring

The deployment pipeline reproduced the audited performance on the same 2026 cohort: AUC 0.826 (0.761–0.885), ECE 0.022, and Brier score 0.065 (Supplementary Table S1). The Youden threshold for the deployed tool was 0.11 (sensitivity 0.640, specificity 0.937). PPV ≥ 99% required a threshold of ≥0.88, and NPV ≥ 95% required ≤0.115. In routine monitoring, 564 spectra from 551 isolates were processed; all 551 isolates and all 564 spectra belong to the 2026 cohort, and the processing period is the same. AUC in this run was 0.838, with a sensitivity of 0.636 and a specificity of 0.951 at the Youden threshold (Supplementary Figure S1). This monitoring analysis confirms that the deployed tool behaves consistently on a subset of the validation cohort; it is not an independent estimate of performance, and the close agreement with the research pipeline (0.838 versus 0.833) should be read in that light.

## 4 Discussion

This screen holds up on the instrument and in the period where it was developed. Everywhere else, it does not. On a laboratory-information-system-audited cohort, evaluated under a strict time split at the isolate level, the model reached an AUC of 0.833 with good calibration (ECE 0.024). That is lower than the 0.909 obtained by cross-validation within the training period, and lower than the 0.90–0.95 typically quoted from random splits. Applied unchanged to external datasets from three other platforms, it lost essentially all discriminative ability (AUC 0.45–0.54). The two results belong together. A model of this kind is tied to the platform and the period on which it was developed, and should not be treated as a portable resistance classifier.

The time-split result tempers earlier expectations. Reports based on random splits and smaller cohorts quoted AUCs of 0.90–0.95, whereas our audited, isolate-level time-split validation yielded a lower but reproducible estimate. Three factors may explain the difference: temporal drift between acquisition periods; the move from spectrum-level to isolate-level evaluation, which removes the optimistic correlation of technical replicates; and the way in which decision thresholds were chosen. The third deserves emphasis because it is easy to overlook. When we derived decision thresholds from the training cohort and applied them unchanged, the sensitivity on the 2026 cohort fell from 0.693 to 0.493. A threshold that achieved an NPV of 0.95 in training achieved only 0.935 in validation. Thresholds chosen on the same cohort used for reporting will therefore flatter a screening model appreciably, and we would encourage future studies to state explicitly whether thresholds were fixed in advance. All three issues apply to MALDI-TOF ML diagnostics in general and deserve explicit reporting in future studies (Varoquaux et al., 2017; Saeb et al., 2017).

Failure to transfer to the external datasets is the second boundary condition, and it needs careful reading. Those cohorts differ from our training data in more than the instrument platform. They also differ in the bacterial population sampled, and, for the KPC and NDM comparisons, in the classification endpoint itself. The design cannot separate these contributions, and we do not claim that the loss of performance is attributable to platform alone. What the analysis does show is operational rather than mechanistic. A pipeline fixed at deployment, applied without modification to spectra from other instruments, did not retain usable discriminative ability. Calibration slopes near zero indicate that its outputs carried no usable probability information either. For a laboratory considering whether an existing tool can be reused on a second instrument, this is the relevant observation, and it is consistent with reports that MALDI-TOF mass accuracy, resolution and laser response differ between platforms (Cuénod et al., 2023; Weis et al., 2020). A design that would separate the contributions would apply one model to the same isolates measured on several platforms; to our knowledge this has not yet been done and it is a clear priority for the field.

Two points follow for practice. The tool is not a stand-alone screen. At any threshold that gives the high negative predictive value a rule-out aid would need, roughly a third of CRKP isolates were still missed. That target was not even reached when the threshold was fixed in advance. Its reasonable use is as a triage aid that shifts the prior probability and prioritizes confirmatory testing, alongside phenotypic susceptibility testing and, where available, carbapenemase typing. Deployment also needs a maintenance plan, and we found none described in this literature. Methods 2.6 therefore sets out explicit operating rules: monitoring of recall, specificity, accuracy and MCC against fixed alert targets, a six-month retraining cycle with immediate retraining on any alert, and verification on a held-out recent period before redeployment. We regard this as a necessary part of any clinical machine-learning deployment rather than an optional add-on.

The main strengths are the LIS-audited cohort of 2,399 isolates, isolate-level evaluation under a strict time split, transparent reporting of calibration, and the explicit separation of thresholds fixed in advance from those chosen after the fact. The labeled spectra and per-spectrum metadata are in MassIVE (MSV000103080), so the estimates reported here can be checked independently rather than taken on trust. The m/z 4517.5–4519.5 cluster, dominant in both cohorts, offers a concrete target for future mechanistic work.

This study has several limitations. First, it is a single-center, single-instrument study. Because the time-split cohort originates from the same institution as training, it tests drift over time but not geographic or epidemiological generalizability. Second, the reference standard is phenotypic susceptibility testing, which is the clinically operative endpoint but would mislabel any isolate that carries a carbapenemase gene without expressing resistance; we did not perform genotyping on the full cohort, so we cannot quantify how often this occurred. Third, the molecular identity of the key spectral peaks is unconfirmed. Fourth, the external cohorts used for transferability testing were not measured on our instrument, their labels were provided by the original authors, and they differ from our training data in population and, in part, in classification endpoint. The transferability result must therefore be read as showing failure of transfer to those datasets as a whole rather than as a measurement of instrument effects; we mitigated comparability by using the same preprocessing and by reporting calibration as well as discrimination. Fifth, the deployed tool’s feature encoding (no square-root transform, 9,554 retained bins) and calibrator (sigmoid) differ from the research pipeline, and we report both on the same cohort for transparency. Sixth, sensitivity is modest at every operating point we examined, and the monitoring analysis overlaps the validation cohort in period and in isolates, so it should not be read as independent corroboration. Finally, the maintenance rules in Methods 2.6 are a reasoned operating procedure rather than an empirically validated schedule. The alert targets are anchored to the 2026 monitoring operating point, and the six-month interval is a default; both will need revision as operational experience accumulates.

## 5 Conclusion

An explainable MALDI-TOF MS machine-learning model, evaluated under a strict time split at the isolate level, reached an AUC of 0.833 for CRKP on the instrument and in the period on which it was developed. Its apparent performance depends materially on how decision thresholds are set. Thresholds fixed in advance on the training cohort gave a sensitivity of 0.493, well below the 0.693 obtained when thresholds were chosen on the validation cohort itself. The model did not retain discriminative ability on external datasets from other platforms, although those datasets also differ in population and label definition, so the loss cannot be attributed to platform alone. Taken together, these results support the use of such a model as a locally developed triage aid rather than as a stand-alone screening test. They also imply that deployment should remain tied to the platform and period of development, under the monitoring and retraining rules set out here. The labeled dataset and per-spectrum metadata are deposited in MassIVE for independent benchmarking, and the deployable tool provides a reference implementation for laboratories using the same platform.

## Data Availability

The datasets presented in this study can be found in online repositories. The names of the repository/repositories and accession number(s) can be found in the article/Supplementary material.

## References

[ref1] Akiba T. Sano S. Yanase T. Ohta T. Koyama M. (2019). “Optuna: a next-generation hyperparameter optimization framework,” in *Proceedings of the 25th ACM SIGKDD International Conference on Knowledge Discovery & Data Mining* (New York: ACM), 2623–2631. doi: 10.1145/3292500.3330701

[ref2] Bizzini A. Durussel C. Bille J. Greub G. Prod'hom G. (2010). Performance of matrix-assisted laser desorption ionization-time of flight mass spectrometry for identification of bacterial strains routinely isolated in a clinical microbiology laboratory. J. Clin. Microbiol. 48, 1549–1554. doi: 10.1128/JCM.01794-09, 20220166 PMC2863943

[ref3] Cai C. Zou M. Chen M. Yuan P. Fang Z. Zhong L. . (2025). Interpretable machine learning of clinical MALDI-TOF spectra discriminates carbapenem-resistant *Klebsiella pneumoniae* while revealing phylogenetic heterogeneity that limits model generalizability. J Mass Spectrom Adv Clin Lab 38, 71–80. doi: 10.1016/j.jmsacl.2025.11.003, 41311462 PMC12648985

[ref4] Calderaro A. Chezzi C. (2024). MALDI-TOF MS: a reliable tool in the real life of the clinical microbiology laboratory. Microorganisms 12:322. doi: 10.3390/microorganisms12020322, 38399726 PMC10892259

[ref5] Cheon D. H. Jang H. Choi Y. K. Oh W. S. Hwang S. Park J. R. . (2025). Clinical evaluation of advanced MALDI-TOF MS for carbapenemase subtyping in gram-negative isolates. J. Clin. Microbiol. 63:e01475–24. doi: 10.1128/jcm.01475-24, 39611795 PMC11784181

[ref6] Clinical and Laboratory Standards Institute (2022). CLSI supplement M100—Performance Standards for Antimicrobial Susceptibility Testing. 32nd Edn. Wayne: Clinical and Laboratory Standards Institute.

[ref7] Cordovana M. Kostrzewa M. Glandorf J. Bienia M. Ambretti S. Pranada A. B. (2018). A full MALDI-based approach to detect plasmid-encoded KPC-producing *Klebsiella pneumoniae*. Front. Microbiol. 9:2854. doi: 10.3389/fmicb.2018.02854, 30542332 PMC6277887

[ref8] Cuénod A. Aerni M. Bagutti C. Bayraktar B. Boz E. S. Carneiro C. B. . (2023). Quality of MALDI-TOF mass spectra in routine diagnostics: results from an international external quality assessment including 36 laboratories from 12 countries using 47 challenging bacterial strains. Clin. Microbiol. Infect. 29, 190–199. doi: 10.1016/j.cmi.2022.05.017, 35623578

[ref9] Gama J. Žliobaitė I. Bifet A. Pechenizkiy M. Bouchachia A. (2014). A survey on concept drift adaptation. ACM Comput. Surv. 46, 1–37. doi: 10.1145/2523813, 40571974 PMC12203853

[ref10] Gato E. Arroyo M. J. Méndez G. Candela A. Rodiño-Janeiro B. K. Fernández J. . (2023). Direct detection of carbapenemase-producing *Klebsiella pneumoniae* by MALDI-TOF analysis of full spectra applying machine learning. J. Clin. Microbiol. 61:e01751–22. doi: 10.1128/jcm.01751-22, 37199638 PMC10281162

[ref11] Harris C. R. Millman K. J. van der Walt S. J. Gommers R. Virtanen P. Cournapeau D. . (2020). Array programming with NumPy. Nature 585, 357–362. doi: 10.1038/s41586-020-2649-2, 32939066 PMC7759461

[ref12] Ke G. Meng Q. Finley T. Wang T. Chen W. Ma W. . (2017). LightGBM: a highly efficient gradient boosting decision tree. Adv. Neural Inf. Process. Syst. 30, 3146–3154.

[ref13] Kumar A. Roberts D. Wood K. E. Light B. Parrillo J. E. Sharma S. . (2006). Duration of hypotension before initiation of effective antimicrobial therapy is the critical determinant of survival in human septic shock. Crit. Care Med. 34, 1589–1596. doi: 10.1097/01.CCM.0000217961.75225.E9, 16625125

[ref14] Lundberg S. M. Lee S. I. (2017). A unified approach to interpreting model predictions. Adv. Neural Inf. Process. Syst. 30, 4765–4774.

[ref15] Marzouk E. Abalkhail A. ALqahtani J. Alsowat K. Alanazi M. Alzaben F. . (2024). Proteome analysis, genetic characterization, and antibiotic resistance patterns of *Klebsiella pneumoniae* clinical isolates. AMB Express 14:54. doi: 10.1186/s13568-024-01710-7, 38722429 PMC11082098

[ref16] Nordmann P. Naas T. Poirel L. (2011). Global spread of carbapenemase-producing Enterobacteriaceae. Emerg. Infect. Dis. 17, 1791–1798. doi: 10.3201/eid1710.110655, 22000347 PMC3310682

[ref17] Pedregosa F. Varoquaux G. Gramfort A. Michel V. Thirion B. Grisel O. . (2011). Scikit-learn: machine learning in Python. J. Mach. Learn. Res. 12, 2825–2830.

[ref18] Pitout J. D. D. Nordmann P. Poirel L. (2015). Carbapenemase-producing *Klebsiella pneumoniae*, a key pathogen set for global nosocomial dominance. Antimicrob. Agents Chemother. 59, 5873–5884. doi: 10.1128/AAC.01019-15, 26169401 PMC4576115

[ref19] Saeb S. Lonini L. Jayaraman A. Mohr D. C. Kording K. P. (2017). The need to approximate the use-case in clinical machine learning. GigaScience 6, 1–9. doi: 10.1093/gigascience/gix019, 28327985 PMC5441397

[ref20] Seng P. Drancourt M. Gouriet F. La Scola B. Fournier P. E. Rolain J. M. . (2009). Ongoing revolution in bacteriology: routine identification of bacteria by matrix-assisted laser desorption ionization time-of-flight mass spectrometry. Clin. Infect. Dis. 49, 543–551. doi: 10.1086/600885, 19583519

[ref21] Singhal N. Kumar M. Kanaujia P. K. Virdi J. S. (2015). MALDI-TOF mass spectrometry: an emerging technology for microbial identification and diagnosis. Front. Microbiol. 6:791. doi: 10.3389/fmicb.2015.00791, 26300860 PMC4525378

[ref22] Tacconelli E. Carrara E. Savoldi A. Harbarth S. Mendelson M. Monnet D. L. . (2018). Discovery, research, and development of new antibiotics: the WHO priority list of antibiotic-resistant bacteria and tuberculosis. Lancet Infect. Dis. 18, 318–327. doi: 10.1016/S1473-3099(17)30753-3, 29276051

[ref23] Tang K. Tang D. Wang Q. Li C. (2021). MALDI-TOF MS platform combined with machine learning to establish a model for rapid identification of methicillin-resistant *Staphylococcus aureus*. J. Microbiol. Methods 180:106109. doi: 10.1016/j.mimet.2020.106109, 33271209

[ref24] Tran N. K. Howard T. Walsh R. Pepper J. Loegering J. Phinney B. . (2021). Novel application of automated machine learning with MALDI-TOF-MS for rapid high-throughput screening of COVID-19: a proof of concept. Sci. Rep. 11:8219. doi: 10.1038/s41598-021-87463-w, 33859233 PMC8050054

[ref25] Varoquaux G. Raamana P. R. Engemann D. A. Hoyos-Idrobo A. Schwartz Y. Thirion B. (2017). Assessing and tuning brain decoders: cross-validation, caveats, and guidelines. NeuroImage 145, 166–179. doi: 10.1016/j.neuroimage.2016.10.038 27989847

[ref26] Virtanen P. Gommers R. Oliphant T. E. Haberland M. Reddy T. Cournapeau D. . (2020). SciPy 1.0: fundamental algorithms for scientific computing in Python. Nat. Methods 17, 261–272. doi: 10.1038/s41592-019-0686-2, 32015543 PMC7056644

[ref27] Wang J. Xia C. Wu Y. Tian X. Zhang K. Wang Z. (2022). Rapid detection of carbapenem-resistant *Klebsiella pneumoniae* using machine learning and MALDI-TOF MS platform. Infect. Drug Resist. 15, 3703–3710. doi: 10.2147/IDR.S367209 PMC9288218 35855758

[ref28] Weis C. Cuénod A. Rieck B. Dubuis O. Graf S. Lang C. . (2022). Direct antimicrobial resistance prediction from clinical MALDI-TOF mass spectra using machine learning. Nat. Med. 28, 164–174. doi: 10.1038/s41591-021-01619-9, 35013613

[ref29] Weis C. V. Jutzeler C. R. Borgwardt K. (2020). Machine learning for microbial identification and antimicrobial susceptibility testing on MALDI-TOF mass spectra: a systematic review. Clin. Microbiol. Infect. 26, 1310–1317. doi: 10.1016/j.cmi.2020.03.014, 32217160

[ref30] Yegorov S. Kadyrova I. Korshukov I. Sultanbekova A. Kolesnikova Y. Barkhanskaya V. . (2024). Application of MALDI-TOF MS and machine learning for the detection of SARS-CoV-2 and non-SARS-CoV-2 respiratory infections. Microbiol. Spectrum 12:e0406823. doi: 10.1128/spectrum.04068-23, 38497716 PMC11064577

[ref31] Yu J. Lin H. H. Tseng K. H. Lin Y. T. Chen W. C. Tien N. . (2023). Prediction of methicillin-resistant Staphylococcus aureus and carbapenem-resistant *Klebsiella pneumoniae* from flagged blood cultures by combining rapid Sepsityper MALDI-TOF mass spectrometry with machine learning. Int. J. Antimicrob. Agents 62:106994. doi: 10.1016/j.ijantimicag.2023.106994, 37802231

