# Supplementary material for: Time-split validation and cross-platform transferability of an explainable MALDI-TOF MS machine-learning model for carbapenem-resistant Klebsiella pneumoniae screening
Source: Front Microbiol. 2026 Oct 28;17:1985869. doi: 10.3389/fmicb.2026.1985869 (PMC13752067; doi:10.3389/fmicb.2026.1985869)
Supplement: Supplementary file 1 [file Table_1.DOCX]

**Supplementary Table S1. Deployment pipeline on the 2026 time-split cohort and routine monitoring.**

| **Metric** | **Value** |
| --- | --- |
| Time-split validation AUC (n = 633) | 0.826 (0.761–0.885) |
| ECE / Brier | 0.022 / 0.065 |
| Youden threshold 0.11: sensitivity / specificity | 0.640 / 0.937 |
| Routine monitoring (n = 564 spectra from 551 isolates): AUC | 0.838 |
| Monitoring at Youden threshold: sensitivity / specificity | 0.636 / 0.951 |
