# Supplementary material for: Time-split validation and cross-platform transferability of an explainable MALDI-TOF MS machine-learning model for carbapenem-resistant Klebsiella pneumoniae screening
Source: Front Microbiol. 2026 Oct 28;17:1985869. doi: 10.3389/fmicb.2026.1985869 (PMC13752067; doi:10.3389/fmicb.2026.1985869)
Supplement: Supplementary file 2 [file Table_2.DOCX]

Supplementary Table S2. Decision thresholds derived from the training cohort and applied unchanged to the 2026 cohort.

Thresholds were derived on the training cohort alone (n = 1,766 isolates) using 5-fold GroupKFold grouped by isolate, with a LightGBM model and isotonic calibrator fitted within each fold; out-of-fold probabilities were averaged within isolate. The resulting thresholds were then applied without modification to the 2026 cohort (n = 633 isolates; 75 CRKP). Training out-of-fold AUC was 0.905; the 2026 cohort AUC was 0.833.

(A) Training cohort, out-of-fold predictions (n = 1,766 isolates)

| **Criterion** | **Threshold** | **Sensitivity** | **Specificity** | **PPV** | **NPV** |
| --- | --- | --- | --- | --- | --- |
| Youden | 0.154 | 0.767 | 0.951 | 0.754 | 0.955 |
| NPV >= 95% | 0.199 | 0.740 | 0.965 | 0.804 | 0.950 |
| PPV >= 99% | 0.885 | 0.438 | 0.999 | 0.992 | 0.901 |

(B) The same thresholds applied unchanged to the 2026 cohort (n = 633 isolates)

| **Criterion** | **Threshold** | **Sensitivity** | **Specificity** | **PPV** | **NPV** |
| --- | --- | --- | --- | --- | --- |
| Youden | 0.154 | 0.493 | 0.962 | 0.638 | 0.934 |
| NPV >= 95% | 0.199 | 0.493 | 0.975 | 0.725 | 0.935 |
| PPV >= 99% | 0.885 | 0.213 | 0.998 | 0.941 | 0.904 |

Note. The threshold targeting an NPV of 95% in the training cohort achieved an observed NPV of 0.935 in the 2026 cohort, i.e. it did not reach the target on the independent cohort. For comparison, thresholds selected descriptively on the 2026 cohort itself yielded a sensitivity of 0.693 at the Youden threshold, whereas the training-derived Youden threshold yielded 0.493 on the same cohort.

PPV, positive predictive value; NPV, negative predictive value; AUC, area under the receiver operating characteristic curve.
